# Wearable magnetic induction-based approach toward 3D motion tracking

**DOI:** 10.1038/s41598-021-98346-5

**Published:** 2021-09-23

**Authors:** Negar Golestani, Mahta Moghaddam

**Affiliations:** Department of Electrical and Computer Engineering, University of Southern California, Los Angeles, CA 90089 USA

**Keywords:** Health care, Engineering, Physics

## Abstract

Activity recognition using wearable sensors has gained popularity due to its wide range of applications, including healthcare, rehabilitation, sports, and senior monitoring. Tracking the body movement in 3D space facilitates behavior recognition in different scenarios. Wearable systems have limited battery capacity, and many critical challenges have to be addressed to gain a trade-off among power consumption, computational complexity, minimizing the effects of environmental interference, and achieving higher tracking accuracy. This work presents a motion tracking system based on magnetic induction (MI) to tackle the challenges and limitations inherent in designing a wireless monitoring system. We integrated a realistic prototype of an MI sensor with machine learning techniques and investigated one-sensor and two-sensor configuration setups for motion reconstruction. This approach is successfully evaluated using measured and synthesized datasets generated by the analytical model of the MI system. The system has an average distance root-mean-squared error (RMSE) error of 3 cm compared to the ground-truth real-world measured data with Kinect.

## Introduction

Over the past decade, monitoring and recognition of human activities have embraced a growing number of practical usages in a broad range of domains such as healthcare, rehabilitation, sports training, virtual reality (VR) gaming, human-computer interface (HCI) systems, finger tracking, daily life-logging, child and elderly care, and assistance for people with cognitive disorders or chronic conditions^1–6^. Tracking and reconstructing limb movements in 3D space facilitates more detailed evaluation, and it is crucial for the analysis and clinical understanding of complex functional movements. Studying biomechanics of human motion has applications in human performance assessment, gesture/posture monitoring, behavioral recognition, gait analysis, and patients’ functionality and improvement evaluation during the rehabilitation period^7–9^.

There are many solutions for tracking body movement using different monitoring sources^10^. Computer vision-based methods, such as Kinect or optical motion capture (MoCap) systems, are the most commonly used techniques that allow users to interact with them and collect data on the user’s motion using depth sensors, color, and infrared cameras^11,12^. However, they inherit computer vision restrictions such as light dependency, coverage limitation, and high computational cost^13,14^. The MoCap systems require an expensive setup of infrared cameras for tracking reflective markers on an individual’s body, which makes them only applicable to the laboratory environment and restricted in physical space. Besides, the markers placement and soft tissue artifacts have a considerable effect on the system accuracy^15,16^. The RF-based solutions are another motion tracking method capturing data based on wireless signal changes (e.g., Doppler frequency shift and signal amplitude fluctuation)^17^. These methods also suffer from environmental dependency and limitation in the number of detectable gestures due to the high cost of training data collection and the lack of multi-user identification capabilities^18^.

Wearable-based solutions are an alternative, cost-effective solution for applications where the optical-based methods are unsuitable. This approach tracks the user’s movement based on the sensors readings placed around the human body^19^. The advancement of sensing technologies, miniaturization, embedded systems, and wireless communication systems combined with predictive models for data analysis and detection have made it possible to develop wearable devices working around the human body for continuous physical activity monitoring. Smart devices like smartphones, smartwatches, and fitness bands are becoming widespread for providing valuable insights about an individual’s performance and health status. These wearables have multiple embedded physiological, inertial, and ambient sensors that enable multi-modal sensing^20^. Many studies have exploited commercial inertial measurement unit (IMU) devices comprised of accelerometers, gyroscopes, and magnetic sensors, for motion tracking based on wearable sensors. An IMU can be attached to a body segment to estimate its movement in space. By combining multiple of them on adjacent body segments, the kinematics of activities can be determined^19^. For example^21^, presents the development of a smart wearable jumpsuit with multiple built-in IMU sensors for automatic posture and movement tracking of infants. The work in^22^ investigates the reliability and validity of IMUs for clinical movement analysis, and^23^ presents a single wrist-worn IMU sensor for high-resolution motor state detection in Parkinson’s disease. Inertial sensing can track limb movements by integrating over sensor measurements, though it is subject to drift since the estimation errors caused by the intrinsic noise can grow unbounded with time^20^.

In some applications, it is possible to achieve improved accuracy and more specific inferences by fusing the subsets of data collected from those sensors compared to single sensor modalities^24,25^. Although these devices provide a solution for physiological health monitoring, condition assessment, and medical diagnosis, they still might face challenges. These single-node devices restrict biosensors’ placement, while optimizing their position can increase the system’s accuracy and robustness in monitoring vital signs (e.g., body temperature and heart rate)^20,26^. Furthermore, in systems relying on data from a single device, variations in position can significantly affect the performance. In motion tracking applications, a single node wearable is not able to cover the entire body. Therefore it cannot get detailed information about the mobility of an individual’s limbs. For example, a smartwatch’s inertial sensor cannot capture the movement of the user’s legs, limiting the system’s ability in classifying activities^20^. A network of distributed wearable devices operating around the human body is an approach that can address these issues.

One of the biggest challenges in wearable-based motion tracking systems is to find the optimum type and number of non-invasive sensors with minimal power consumption to achieve acceptable accuracy and satisfy guidelines and constraints. In^20^, we introduced magnetic induction-based human activity recognition (MI-HAR), a wireless system based on magnetic induction combined with classification techniques to detect human activities. The proof-of-concept for the proposed system is also provided by considering a conceptual system with eight on-body sensors distributed on a user’s body. We synthesized sensors data for several activities using a calibrated magnetic induction (MI) system model^20,27^ and two publicly available MoCap datasets. The accuracy of the analytical MI model in generating time-series MI data using 3D motion data is verified by experimental measurements. Furthermore, the system performance is evaluated using several machine learning algorithms and deep learning frameworks classifying human actions based on recorded MI data. We showed that this system can address challenges in terms of power consumption, accuracy, coverage, privacy, and cost. In this work, we extend the MI-HAR system and investigate the capability of the MI system in 3D motion tracking instead of identifying human activity. Moreover, we build and integrate a realistic prototype of an MI sensor with regression models and study one-sensor and two-sensors configuration setups for motion reconstruction. The MI model and Variational Auto-Encoder (VAE) are also employed to generate synthetic data for training regressors without the need for measured data. The trained model is then evaluated on real-world measurements to show that the system has an acceptable dynamic spatial resolution in tracking sensor movements compared to the ground truth motion captured by Kinect.

## Results

### Operating principle

The MI-based communication system is a short-range wireless physical layer that transmits signals by inductive coupling between the wire coils rather than radiating as is done in conventional methods^27,28^. The transmitter node uses a coil to produce an oscillating magnetic field at a specific frequency. Due to the small radiation resistance of the coil, a negligible amount of energy propagates to the far-field. It removes the multipath fading effect resulting in a better quality of service (QoS) compared to conventional propagating wave systems^29^. Each sensor node’s (receiver) main component is a coil, which is lightweight, portable, inexpensive, simple, and wearable to capture the transmitter’s generated magnetic field. An MI sensor module can be manufactured for less than $20, compared to expensive sensors such as Bluetooth IMU with an average cost of $100^20^. The MI system experiences much less energy absorption in lossy dielectric media (e.g., human body) compared to conventional radio-wave propagation technologies, and therefore can transmit a signal with much less power for the same range. The signal also remains in a ‘bubble’ around the coil, which minimizes the leakage outside the targeted coverage range, reduces interference, increases security, and provides a personalized space for the user. These characteristics make the MI system power-efficient compared to other short-range communication systems such as Bluetooth^20,30–32^.

According to Faraday’s law, the time-varying magnetic field induces a voltage in sensor nodes proportional to the rate of magnetic flux change through their coils. For a predefined coil geometry and operating frequency below 30 MHz, where the environmental effects are negligible, the flux change rate is a function of the sensor coils’ position, and orientation relative to the transmitter^20,27^. The relationship function from spatial data into induced voltage is non-linear and surjective, and the tracking problem objective is to estimate the sensors’ positions given the induced voltage measurements.

### System architecture

We used an analytical model of the MI system presented in^20,27,33^ to calculate the induced voltage at each sensor coil given its position and orientation. This forms the basis of the data-driven backward estimation algorithm that retrieves a node’s position using its observed data. It helps assess the system performance under different configurations, such as changing the number or arrangement of sensor coils to find the near-optimal setup with acceptable tracking accuracy. Since the model is a function of relative distance and alignment of coils to the transmitter, we transform the coordinate system to locate the new coordinate system’s origin at the center of the transmitter coil, with the coil’s surface normal oriented in the Z direction. Given the sensors’ spatial data, we compute the coordinate transformation matrix and calculate each coil’s position and orientation in the new coordinate frame. We explored the node’s position $p=(x,y,z)$ with the resolution of 1 cm, and alignment ${\hat{n}}=(\sin \theta \cos \phi ,\, \sin \theta \sin \phi ,\, \cos \theta )$ with the resolution of 5° as these resolutions are expected to satisfy the accuracy requirements for motion tracking applications^34,35^. It also provides enough data points within the search domain for comprehensive performance analysis of the system with different configuration settings. The possible solutions, which are a unique single-point in an optimal configuration, are retrieved for a given set of observed data. The domain of search space for both the center of a coil and its surface normal alignment is defined as follows. The search domains for the *xyz* parameters are set such as to represent the average ranges of distances where sensors can be placed for both male and female subjects relative to an on-body central node on their torso. The $\theta$ and $\phi$ parameters generate the coil’s alignment, and therefore their search domains are defined such that it is possible to describe rotations for the coil that do not result in values close to zero.

1 $$\begin{aligned} \begin{array}{cc} \begin{array}{cc} x &{} \, \in \, [-20\text { cm}, \, 20 \text { cm} \,] \\ y &{} \, \in \, [-20\text { cm}, \, 20 \text { cm} \,] \\ z &{} \, \in \, [-60\text { cm}, \, -10 \text { cm} \,] \\ \end{array} &{} \qquad \qquad \begin{array}{cc} \theta &{} \, \in \, [\, 0^\circ , \, 60^\circ \,] \\ \phi &{} \, \in \, [\,0^\circ , \, 360^\circ \,] \\ \end{array} \end{array} \end{aligned}$$

**Figure 1:**
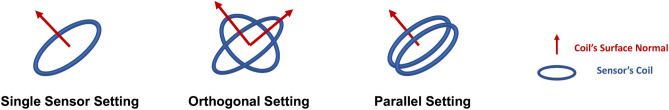
Configuration Settings. Schematic representation of configuration settings employed in the experiments.

**Figure 2:**
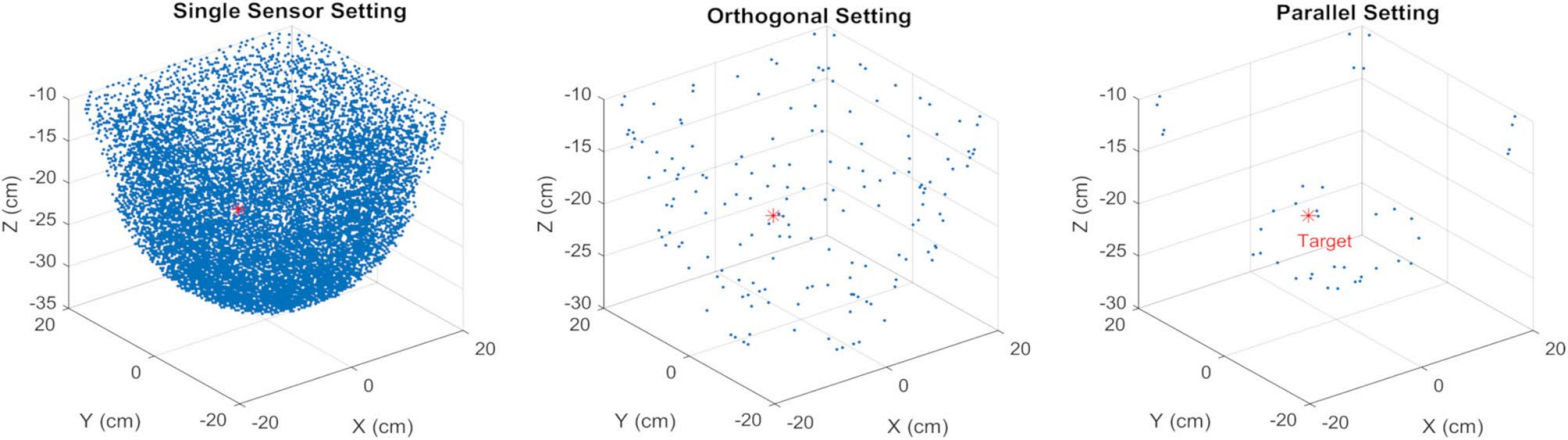
Data-driven location tracking. Estimation of a predefined target point across all settings, where each point represents a possible node’s position with at least one alignment that can produce the given set of inputs for the defined setting.

We studied the performance of an MI sensor (single sensor setting), where the coil can be aligned in any direction. We also adopted two-sensor configurations and investigated different alignment setups. Among these setups, we present the performance analysis of setups where coils’ surface normal are aligned in the same direction (parallel setting) or perpendicular to each other (orthogonal setting). Figure 1 depicts the configuration of sensors in each described setting. In these experiments, the induced voltage measured at the coils is used as input for location estimation. Figure 2 shows an example result of the data-driven backward estimation algorithm. As the results display, there are many possible solutions for a single sensor setup, and this number reduces by adding another sensor. The sensor voltage data are assumed to be measured with 1 mv accuracy and given as inputs to the algorithm. A comparison between the two-sensor configurations shows that the parallel setting outperforms the orthogonal setting. Although a unique solution cannot be returned as an output, results suggest that the regression methods with proper constraints can meet the minimum required accuracy for position tracking.

### Data collection

We designed and built an MI sensor for 3D motion tracking (see “Methods” section), representing the movements by variation in the MI signals received from the transmitter instead of measuring spatial data via conventional sensors such as IMUs. To evaluate the capability of the proposed MI sensor, we employed regression algorithms and investigated their performance on the MI sensor’s data. Validating and testing machine learning methods is critical and challenging due to the difficulty of collecting realistic valid data and the lack of labeled data. One solution is to create synthetic data for training the model, and here, we used a VAE model to produce time-series motion data. The MI data corresponding to the synthetic movements are then generated using an analytical MI system model^20,27^. The regressors are then trained on these synthesized data, which removes the need for supervised training measured data. A point to consider is that the MI system model must be calibrated only once to scale the synthetic training data to sensor measurements and tune the regression algorithm (see “Methods” section). The trained machine learning regressors on the synthetic data are then tested on real-world measurements and reported for comparison.

### Evaluation

We deployed machine learning regression algorithms to solve the inverse problem of estimating a node’s 3D position (x,y,z) from its sensors’ measurements in meters. The performance of several regression models, including extra trees (ET), random forest (RF), K-nearest neighbors (KNN), , light gradient boosting machine (LightGBM), multi-layer perceptrons (MLP), decision trees (DT), and linear regression (LR) is compared using PyCaret^36^, an open-source machine learning library in Python. The models are trained on 70% of synthetic data and then scored on the remaining data using the 10-fold cross-validation method. The metrics used for comparison are RMSE, mean absolute percentage error (MAPE), and R-squared (R^2^). Before fetching data into the regressors, each feature is standardized individually, and the missing values are substituted with previous non-missing values. The processed data are then divided into fixed-length segments of 2 s using the sliding window technique with a 0.1 s step size.

Table 1 summarizes the performance results of all models on the synthetic data for different settings. As the results show, the moving node’s distance and position in the Z-direction with respect to the transmitter coordinate frame can be tracked with competing accuracy compared to other methods using wearable sensors (e.g., accelerometer) for motion tracking^19,35^. All of the results and metrics on motion tracking are reported in units of meters, and the best scores are denoted in bold. It is worth mentioning that the mutual inductance between two coils varies as their distance, lateral alignment, or angular alignment changes. As we assume that the transmitter coil is centered at the origin and aligned in the Z direction, any movement in the X or Y direction results in a similar lateral misalignment and consequently the same path loss. This characteristic makes it challenging for the regression model to estimate MI sensors’ location accurately and differentiate between motion in the X and Y directions. Because the method is able to estimate the distance and location in the Z direction with good accuracy, adding another transmitter with an antenna surface orthogonal to the primary antenna enables the node’s motion tracking in the new direction (e.g., X), resulting in 3D positional tracking. However, the dual-transmitter setup can drain power at twice the rate of a single-transmitter system, which can be addressed with proper design modifications. For example, time-division multiplexing (TDM) or frequency-division multiplexing (FDM) approaches can be adopted as low complexity hardware techniques using a single transmitter to reduce the power, area, and cost of a dual-antenna system instead of using two separate transmitters. Then the receiver sensor can record transmitted signals from two perpendicularly aligned antennas, which provides adequate data for tracking its location in 3D. This system keeps power consumption the same as a single-antenna system, while increasing tracking accuracy in all three dimensions.

**Table 1:** Performance summary.

	**Model**	Distance			X			Y			Z		
		RMSE	MAPE	R^2^	RMSE	MAPE	R^2^	RMSE	MAPE	R^2^	RMSE	MAPE	R^2^
Single Sensor	ET	**0.028**	0.043	0.898	0.05	6.548	0.232	0.035	**3.877**	0.102	0.031	0.05	0.868
	RF	**0.028**	**0.042**	**0.9**	0.05	6.872	0.25	0.035	3.88	0.125	0.031	**0.049**	0.871
	KNN	0.029	0.044	0.89	0.052	**6.155**	0.161	0.037	3.923	0.017	**0.033**	0.052	**0.856**
	**LightGBM**	**0.028**	**0.042**	**0.9**	**0.048**	6.873	**0.29**	**0.034**	3.931	**0.173**	0.031	**0.049**	0.872
	MLP	0.03	0.045	0.883	0.049	6.806	0.278	**0.034**	3.999	0.147	0.034	0.053	0.846
	DT	0.039	0.057	0.807	0.069	10.529	− 0.45	0.049	4.05	− 0.708	0.043	0.067	0.753
	LR	0.04	0.066	0.796	0.052	8.079	0.177	0.035	4.095	0.138	0.041	0.069	0.769
Orthogonal	**ET**	**0.022**	**0.029**	**0.879**	**0.04**	**4.897**	**0.537**	**0.011**	**0.851**	0.18	**0.005**	**0.007**	0.169
	RF	0.023	0.031	0.863	0.043	5.266	0.47	0.031	2.53	0.362	0.026	0.037	0.827
	KNN	**0.022**	**0.029**	0.875	0.042	5.749	0.487	0.03	2.137	**0.387**	0.025	0.034	**0.84**
	LightGBM	0.026	0.036	0.826	0.05	5.989	0.294	0.035	3.018	0.158	0.029	0.043	0.779
	MLP	0.026	0.037	0.823	0.05	5.239	0.277	0.036	3.191	0.134	0.03	0.043	0.774
	DT	0.033	0.041	0.718	0.062	7.04	− 0.096	0.044	2.71	− 0.313	0.037	0.049	0.642
	LR	0.035	0.05	0.688	0.053	6.829	0.184	0.037	3.2	0.103	0.038	0.057	0.625
Parallel	**ET**	**0.011**	**0.018**	**0.947**	**0.037**	**7.539**	**0.365**	**0.028**	5.184	**0.23**	**0.014**	**0.025**	**0.917**
	RF	**0.011**	0.019	0.94	**0.037**	9.49	0.334	**0.028**	5.471	0.193	**0.014**	0.026	0.908
	KNN	0.012	0.021	0.932	**0.037**	10.543	0.352	**0.028**	**4.855**	0.196	0.015	0.027	0.9
	LightGBM	0.012	0.021	0.932	0.04	10.004	0.261	0.03	5.827	0.075	0.015	0.028	0.897
	MLP	0.013	0.022	0.924	0.04	9.251	0.231	0.031	6.268	0.022	0.016	0.03	0.881
	DT	0.016	0.026	0.882	0.053	14.302	− 0.338	0.04	5.478	− 0.636	0.02	0.035	0.818
	LR	0.021	0.037	0.804	0.041	11.848	0.185	0.031	6.015	0.048	0.023	0.043	0.762

Results of motion tracking algorithms on the synthetic data for different settings.

**Figure 3:**
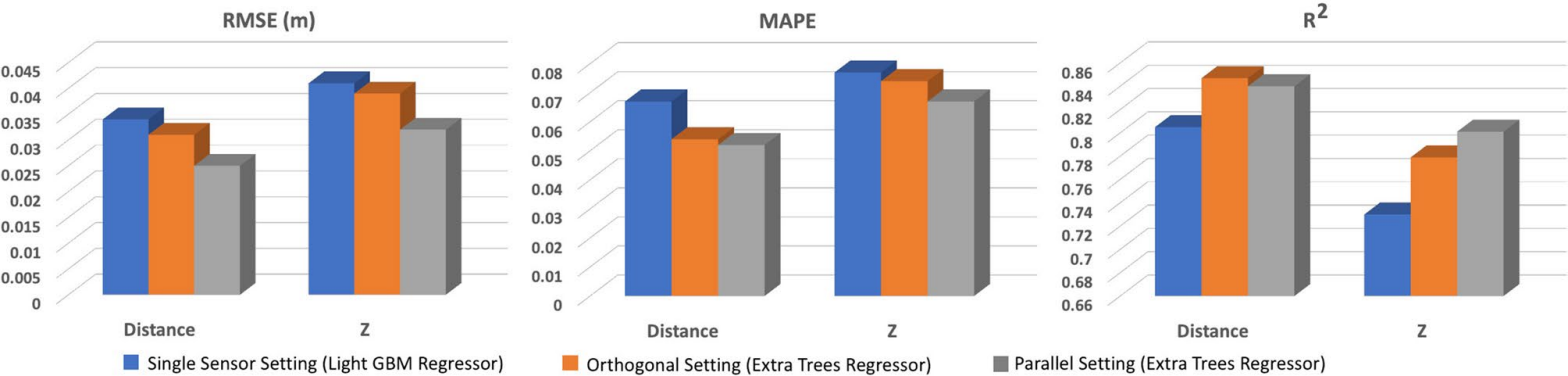
Performance on measured data. Results of motion tracking methods in the 3D space domain using real-world data for different settings.

**Figure 4:**
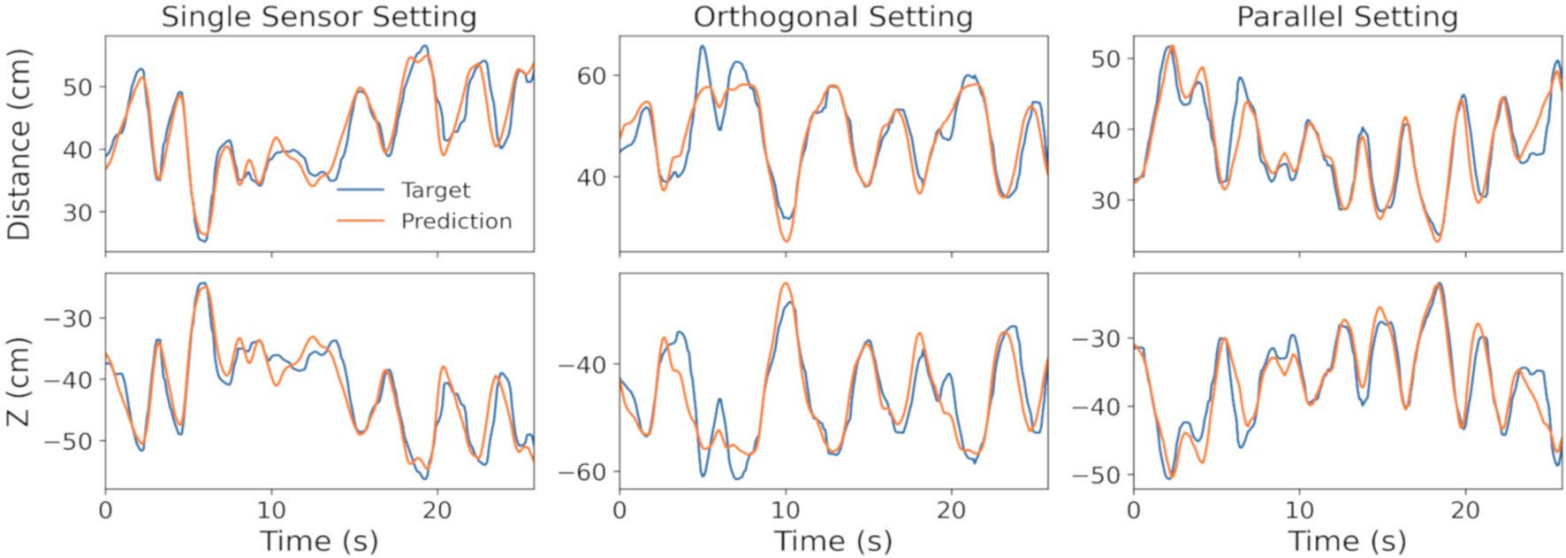
Measurement versus estimation. The measured ground-truth and estimated distance and Z-direction displacement of MI sensors relative to the central node over time.

Among the research studies on 3D motion tracking, the work in^37^, for example, has reported results on tracking subjects’ arm motion using smartwatch IMU data. The results show that the system can achieve the highest accuracy when the torso is static, with a median error of 8.8 cm. Moreover,^38^ presents a framework for reconstructing human motion with the highest accuracy of 6 cm using four 3D accelerometers attached to the user. The work in^39^ has proposed the utilization of spinning linearly polarized antennas to track translation of an object attached to a passive radio frequency identification (RFID) tag array in 3D and has reported an average error of 13.6 cm. To provide a realistic assessment of real-world performance, we evaluated each of the optimal models’ tracking accuracies on measured data as well. According to the score measures reported on synthetic data, the LightGBM regressor in the single-sensor setting and the ET regressor in the two-sensor (orthogonal and parallel) settings outperform other models. Figure 3 presents the evaluation measures of optimal models using the measured data for each setting. Representative samples of motion tracking in all settings are also displayed in Fig. 4. Our results indicate that the parallel setting with the optimal regression model outperforms other settings on both measured and synthetic MI data.

## Discussion

We proposed a 3D motion tracking system based on magnetic induction and provided a proof of concept by experimental measurements conducted using off-the-shelf devices and prototypes. We employed an HF RFID transmitter module equipped with a loop antenna and an MI sensor as the central node and receiver, respectively. The designed sensor is a simple integrated circuit equipped with an Arduino to record the samples of received signals from the transmitter. To implement the proposed system for real-world applications, proper modifications should be taken into account. For example, the MI coils should be designed to be suitable for wearing on the human wrist, arm, and ankle. Furthermore, a wearable custom-designed central node capable of driving a controlled amount of current at the operating frequency through its coil is required. The receivers should cover the range of about 0.5 m to 1 m with minimum power consumption.

The RF output power of the reader used in this work is 1 Watt, which can be reduced by designing a customized MI system capable of communication and data transmission with high accuracy. The reader sends continuous sine waves while the sensor records samples of received power. However, a customized MI transmitter (central node) operating with pulsed shape sine wave signals can achieve similar accuracy within the targeted coverage range at significantly lower power. Determining optimal pulse rate and sampling rate plays a critical role in designing a power-efficient high-accuracy MI-based motion tracking system. Hardware development at the sensor side is another factor that affects system performance. For example, impedance matching reduces power losses and consequently enhances the system transfer efficiency and gain. There are research studies focused on details of designing low-power MI-based communication systems. The research work presented in^40^ proposes a transceiver design exploiting the low path loss of Magnetic Human Body Communication (mHBC) communication channels toward ultra-efficient body area networking. The transmitter and receiver, respectively, require only 7.15 and 4.7 pJ/bit for communication within the range close to the required coverage range in our application. Their design is a helpful reference for implementing MI transceivers.

Another approach for realizing the system with lower power requirements is reducing the number of nodes with batteries. One implementation strategy is to make the central node serve as both transmitter and receiver. It means that the central unit can broadcast the signal and listen back to the responses reflected from the sensors, similar to an RFID system based on passive (battery-less) tags. In an RFID system, the reader sends an interrogation signal to the transponders, which is also used to energize the tag. The tag activates and sends back its unique identifier (UID) if the received power is higher than its sensitivity^41^. A modulation resistance connected in parallel with the tag antenna switches between two different (usually conjugate matching and a short circuit) load impedances at the clock rate of the signal transmitted from the reader to modulate the backscattered signal^42^. Therefore, the central node can communicate with the tags via a secure near-field link backscattering from them. The amplitude of the demodulated signal is calculated and reported at the reader side by a value proportional to the received signal’s power level, known as the received signal strength indicator (RSSI). A point to consider is that load modulation is not a practical solution for data transmission in an MI-based motion tracking system. The reason is that the backscattered field, and consequently, the voltage signal received by the reader, switches over two values^12^. The average power returned to the reader is no longer a direct function of distance and misalignment between coils since it varies by the number of zeros and ones in the data stream. Therefore, proper modulation and modifications are required to be able to employ existing RFID protocols.

Here we have compared the relationship between RSSI and MI signals with motion data by recording RSSI data of RFID tags in addition to the MI-sensor data. The experiments are performed using a framework similar to the setup explained for MI measurements (see “Methods” section) using HF RFID tags instead of MI sensors. We employed custom air-cored, three-layer copper coils with a 5 cm radius and 34 American wire gauge (AWG) wire diameter as the tag antenna attached to STMicroelectronics ST25DV04K RFID tag. We measured motion and RSSI data of RFID tags reported from the reader for 112 experiments. The best calculated average R^2^ and the correlation between RSSI and the distance of the tag from the reader are respectively 0.11 and 0.33. For an MI sensor, the calculated R^2^ and correlation over 220 samples are 0.61 and 0.78, respectively. These results indicate that the MI signal has a stronger relationship with its motion compared to a passive tag (see Supplementary Information).

## Methods

### Hardware design

The system consists of a transmitter (central) node generating an oscillating signal at 13.56 MHz. We used ISC.LRM1002 long-range RFID reader module^43^ attached to ISC.ANT310/310 long-range HF antenna^43^ to generate the RF signal. Since we used this setup for RFID measurements presented in the discussion, we used the same transmitter for a better comparison. The receiver node consists of MI sensors. Each sensor includes an air-cored, single-layer copper coil with a 5 cm radius and 10 AWG wire diameter to capture the transmitter’s signal and measure the induced voltage. Resistance and self-inductance of the coil measured by vector network analyzer (VNA) at the resonance frequency are 101 m$\Omega$, and 241 nH, respectively. To improve the system efficiency, we have employed resonant inductive coupling attached to the coil. The tuning circuit can be as simple as a capacitor to tune the frequency or be a $\Pi$ or T matching circuit to tune the frequency, control Q-factor, and match input and output impedances for higher power transfer^44^. Here, we used a 560 pF capacitor parallel to a trimmable capacitor with an adjustable range of 3–10 pF to accurately tune the circuit to resonance.

**Figure 5:**
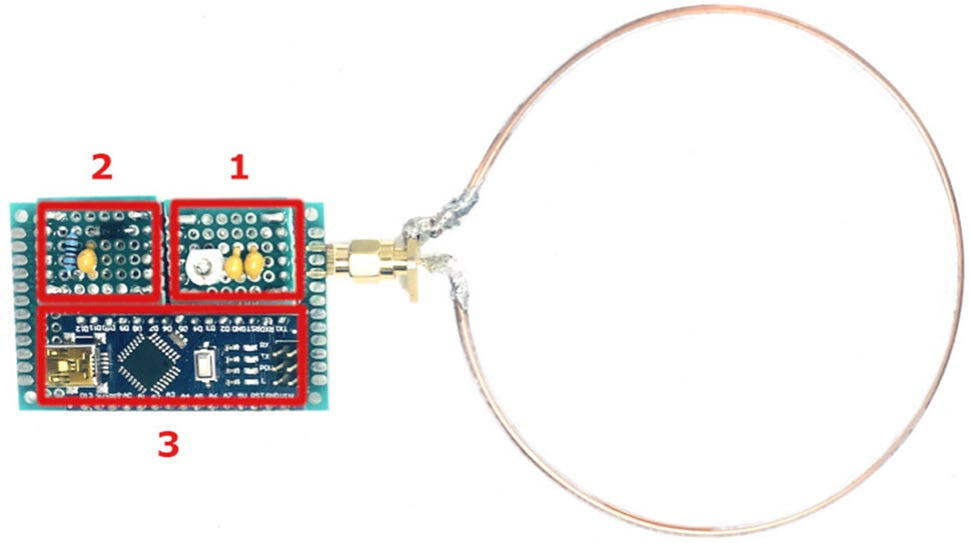
MI sensor prototype hardware. The prototype consists of three main parts: (1) Variable capacitor for frequency tuning, (2) Envelope detector, (3) Arduino microcontroller for measurement.

The transmitted AC signal attenuates as a function of distance and alignment of the node with respect to the transmitter antenna. To track the signal’s amplitude changes, we used an envelope detector consisting of an IN5817 Schottky diode, a resistor of 1 K$\Omega$, and a capacitor of 1 nF. The envelope detector’s output, which is the resistor’s voltage, is measured by an Arduino Nano (ATmega168) microcontroller. The resolution of ADC (analog pin A1) is 10 bits for a defined measurement range. Figure 5 depicts MI sensor components.

### Measurements

We employed a Microsoft Kinect v2 to capture the 3D position and alignment of the transmitter and the MI sensor node. The Kinect sensor consists of a depth camera, an RGB camera, and a microphone array sensor. The RGB camera and depth camera respectively provide 1920 × 1080 color image and 512 × 424 depth image at 30 frames per second with a resolution of a few millimeters in measure range between 0.5 m to 4.5 m^45^. The depth stream provides the sensor’s distance to every point within its area of coverage. As the cameras have different pixel resolutions and are not perfectly aligned, three coordinate spaces and types are defined: color space point $(x_c, y_c)$, depth space point $(x_d, y_d)$, and camera space point $(x_w, y_w, z_w)$, representing a point in the color images, depth images, and real-world, respectively. The software development kit (SDK)’s mapping function can be used to map a point from one coordinate space to another.

We used colored markers to facilitate motion tracking of the devices and developed a video processing algorithm analyzing the color frames to locate pixels corresponding to the target color. The transmitter antenna and the MI node are labeled with distinct colored markers and placed in front of a white background. A threshold range is set for each color to extract pixels with the color value within the defined range. The detected pixels are classified to $N_m$ clusters, where $N_m$ is the number of markers, using K-means clustering methods. Then, the connected neighboring pixels of each cluster are grouped. Since the markers are colored foam balls, the circle with the minimum area enclosing each set is calculated, and the largest region is given as the target circle. The next step is mapping color to camera space to find the corresponding spatial location of each extracted color pixel. The result is a list of 3D real-world points mapped from the target circle’s pixels, and each marker’s location is computed by taking the median over all the calculated values. This process repeats for each new color frame that Kinect captures.

The analytical model requires the center and alignment of the transmitter and receiver coils/antennas as inputs to estimate the induced voltage. To determine a coil’s surface normal, at least three markers ($M_i:i\in \{1,\ldots ,N_m\} \text {\, with }N_m>=2$) are required. Hence, we used four red and three blue markers to track the transmitter antenna and the MI sensor node. The center of each device is calculated by averaging over its markers’ location $c=\sum _{i=1}^{N_m} M_i$, and its surface normal is also calculated by the cross product of vectors passing through the markers: ${\hat{n}}=\mathbf {v}_1 \times \mathbf {v}_2$ where $\mathbf {v}_1=M_1-M_2, \, \mathbf {v}_2=M_1-M_3$. We applied the median filter, a non-linear digital filtering technique, to remove noise and spikes in the extracted location and alignment data.

**Figure 6:**
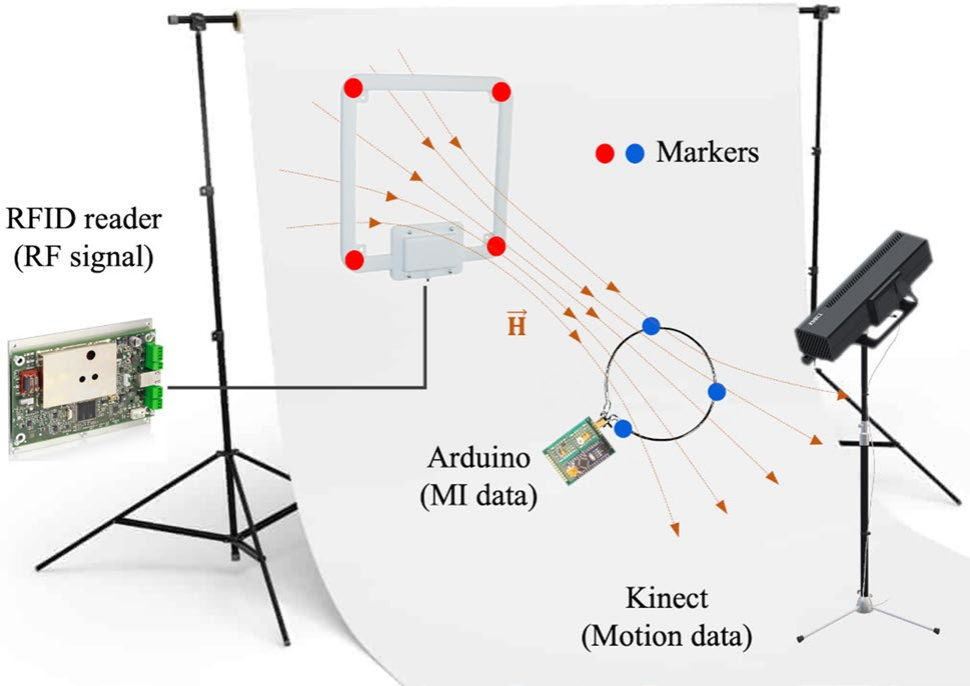
Measurements. Schematic representation of measurement setup, including motion capture and sensor data collection.

The induced voltage, $V_{ind}$, at the MI sensors is measured for 30 s via Arduino by using a Python script that controls the recording in order to synchronize Kinect’s motion data and Arduino’s measurements. The sampling frequency is 100 Hz, and the reference voltage range is 0 V to 5 V, which results in the quantization interval of 5/1024 V. The data streams of the node’s MI sensors are recorded and used as inputs for the regression model to estimate the device’s location. The sampling rate of motion data recorded by Kinect and the sensors’ data are different. Therefore, all recordings are resampled with a sampling interval of 100 ms, which also handles the missing sample values. The measurement setup of experimental measurements is presented in Fig. 6.

### Synthetic data

A VAE is based on the auto-encoder architecture and is composed of encoder and decoder networks. The encoder compresses the data into a lower-dimensional space called the latent space representation. The decoder decompresses the reduced representation code to reconstruct the original data. The VAE learns the probabilistic interpretation of these networks and generates new samples using different latent variables as input. Consider dataset $\{\, x^{(i)} \, \}_{i=1}^N$ that consists of *N* i.i.d. samples of some variable ${\mathbf {x}}$. VAEs assume that the data are generated by a random process with continuous latent variable, and each latent variable ${\mathbf {z}}$ is related to its corresponding observation ${\mathbf {x}}$ through likelihood $p_\theta ({\mathbf {x}}|{\mathbf {z}})$, where $p_\theta$ is a probability distribution with parameters $\theta$. This probabilistic interpretation of the decoder can decode a latent (hidden) representation code into a distribution over the observation. Similarly, the encoder network returns a latent code sampled from the posterior density distribution $p_\theta ({\mathbf {z}}|{\mathbf {x}})$ given a sample from the data space^46^. While both prior $p({\mathbf {z}})$ and likelihood $p({\mathbf {x}}|{\mathbf {z}})$ can be formulated exactly, the posterior $p({\mathbf {z}}|{\mathbf {x}})$ requires an intractable integral over the latent space. Hence, an approximate posterior $q_\phi ({\mathbf {z}}|{\mathbf {x}})$ closest in Kullback-Leibler (KL) divergence to the actual, intractable posterior distribution is considered. The approximate posterior is parameterized by variational parameters $\phi$, and the training objective is a tractable lower bound to the log-likelihood^47^:

2 $$\begin{aligned} \log p(x) \ge {\mathbb {E}}_{q_\phi ({\mathbf {z}}|{\mathbf {x}}) }\; \Big [ \, \log \frac{p_\theta ({\mathbf {x}}, {\mathbf {z}})}{q_\phi ({\mathbf {z}}| {\mathbf {x}})} \, \Big ] = {\mathscr {L}} ({\mathbf {x}};\theta ,\phi ) \end{aligned}$$

and can be equivalently written as:

3 $$\begin{aligned} {\mathscr {L}} ({\mathbf {x}};\theta ,\phi ) = {\mathbb {E}}_{\, q_\phi ({\mathbf {z}}|{\mathbf {x}})} \, \big [ \, \log p_\theta \, ({\mathbf {x}}| {\mathbf {z}}) \, \big ] \, - {\mathscr {D}}_{KL} \big ( \, q_\phi ({\mathbf {z}}| {\mathbf {x}}) \, || \, P_\theta ({\mathbf {z}}) \, \big ) \qquad \end{aligned}$$

On the right-hand side of equation (3), the first term, reconstruction error, represents the likelihood of the model reconstructing the input data. The second term, variational regularization term, is the KL divergence and makes the approximate posterior $q_\phi ({\mathbf {z}}| {\mathbf {x}})$ to be close to $p_\theta ({\mathbf {z}})$. The ${\mathscr {L}} ({\mathbf {x}};\theta ,\phi )$ is a lower bound on the log probability of data $p_\theta ({\mathbf {x}})$, evidence lower bound (ELBO). Maximizing ELBO with respect to the model parameters $\theta$ and variational parameters $\phi$ respectively maximizes the marginal probability $p_\theta ({\mathbf {x}})$ and minimizes the KL divergence^46^.

We trained the VAE model using the sensors’ motion data tracked by the Kinect to produce synthetic time-series samples. After training the model, new time-series data can be generated by sampling from latent space ${\mathbf {z}}$ with normal distribution parametrized by the mean and the variance^47^. The generated data include the motion of the coils’ center and alignment in 3D space for a predefined sensor setting. We synthesized angular variables $\theta$ and $\phi$ to calculate the corresponding coil’s surface normal ${\hat{n}}$ that can be defined as ${\hat{n}} = (\sin \theta \, \cos \phi , \; \sin \theta \, \sin \phi , \; \cos \theta )$, where the variables $\theta$ and $\phi$ can take values in the range of 0-90 and 0-360 degrees, respectively.

**Figure 7:**
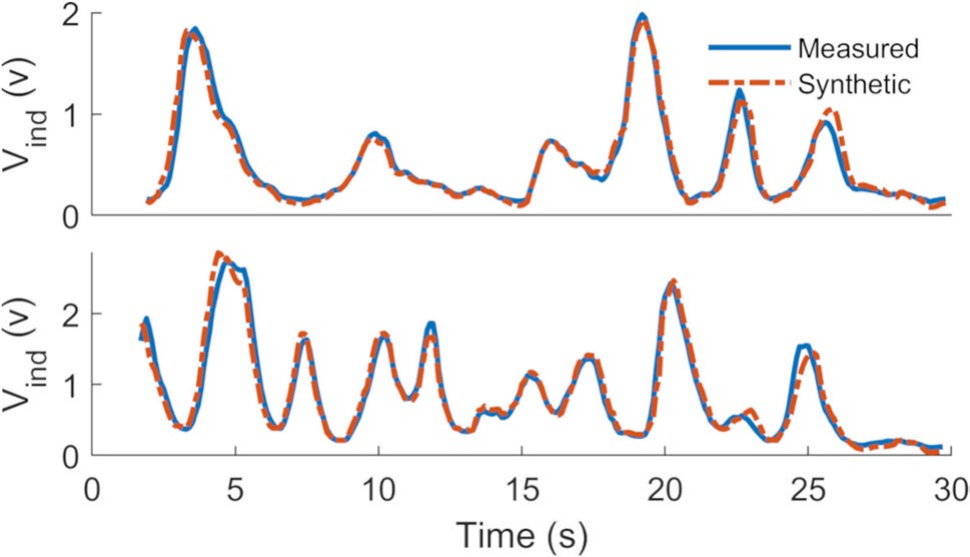
Measurement versus synthetic data. The measured and simulated induced voltage at the MI sensor during two different arbitrary movements, such that both relative alignment and location of the coil vary relative to the central node.

We have performed the experiment for 220 motions, including spatial translation and rotation ($N_s$=220). The measured motion data samples of these experiments are used for training VAE to generate synthetic motion data. Then their corresponding MI signal is estimated using the two-port network model of the MI system^20,27^ given node motion data. To evaluate the performance of the analytical model, we fetched the captured motion data by the Kinect system as input and estimated the corresponding induced voltage at the MI sensors for each measurement experiment. The circuit model is calibrated by finding the scale and bias of the synthesized data with respect to the measurements. Considering $s_i$ and $m_i$ as the generated synthetic data and measurements corresponding to a motion sample, the scale $a = \frac{1}{N_s} \, \sum _{i=1}^{N_s} \, \frac{\sigma _{mi}}{\sigma _{si}}$ and bias $b = \frac{1}{N_s} \, \sum _{i=1}^{N_s} \, \mu _{mi} -\frac{\sigma _{mi}}{\sigma _{si}}\, \mu _{si}$ can be calculated, where $\mu _{si} \, ,\, \sigma _{si} \,,\, \mu _{mi} \, , \, \sigma _{mi}$ represent the mean and standard deviation of synthetic data and measurements corresponding to the *i*th motion sample from $N_s$ samples. Figure 7 shows the measured and simulated sensors’ data during their movement, taken from the evaluation dataset after calibrating the model.

**Figure 8:**
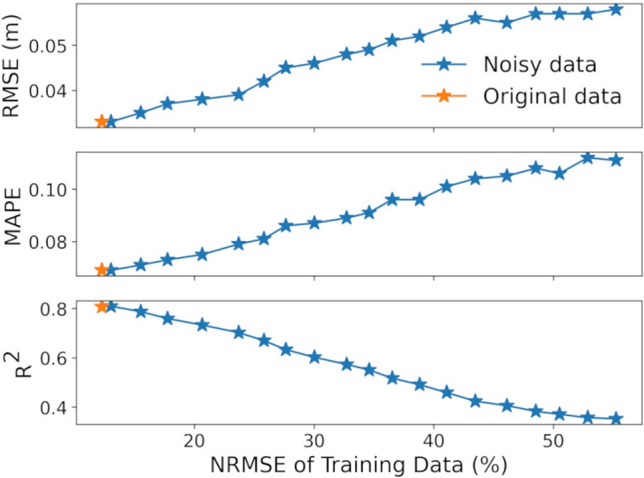
Error evaluation. Performance of motion tracking algorithm trained on noisy data with different normalized root mean square error (NRMSE) values.

The average normalized root-mean-squared error (NRMSE) and cross-correlation of the synthesized and measured data for all experiments are 12% and 0.91, respectively. It should be noted that the reported metrics consider not only the MI system model inaccuracy but also the error associated with the Kinect-based marker tracking algorithm and Arduino measurements. The variation between the real-world and synthetic samples affects the performance of the motion tracking algorithm that trains on the synthetic MI data. We re-assessed the performance of the regression model trained on noisy synthetic datasets to provide an evaluation of errors caused by the analytical MI system model in motion tracking. We considered the single sensor setting and its corresponding optimal regression model LightGBM for the analysis. Gaussian noise with zero mean and standard deviation of $\sigma$ varying between 0 to 1 is added to the data generated by the MI model. The resulting datasets are separately given to a pre-trained regression model for training and then evaluated on the measured samples. The NRMSE value for each noisy dataset is calculated by comparing measured data and their corresponding noisy synthetic data. Figure 8 displays the performance of a machine learning regressor in motion tracking trained on these datasets with different NRMSE values (noise levels). The results show the effect of the MI model in generating realistic samples on the performance of the motion tracking algorithm.

## Supplementary Information

Supplementary Information 1.

Supplementary Information 2.

Supplementary Information 3.

Supplementary Information 4.

Supplementary Information 5.

## Data Availability

The datasets generated during and/or analyzed during the current study are available from the corresponding author on reasonable request.
