# Supplementary material for: Wearable magnetic induction-based approach toward 3D motion tracking
Source: Sci Rep. 2021 Sep 23;11:18905. doi: 10.1038/s41598-021-98346-5 (PMC8460632; doi:10.1038/s41598-021-98346-5)
Supplement: Supplementary file 1 — Supplementary Information 1. [file 41598_2021_98346_MOESM1_ESM.pdf]

Supplementary Information

RFID Measurement Setup

Industrial passive tags are usually built as integrated circuits (ICs), which can be attached to a customized antenna. Here we adopted the ST25DV04K chip manufactured by STMicroelectronics, which is a dynamic near-field communication (NFC) radio frequency identification (RFID) tag with 4 Kbit electrically erasable programmable memory (EEPROM) and fast transfer mode (FTM) capability. This dynamic tag uses ISO 15693 and ISO 14443 protocols based on passive RFID technology and operates in the high frequency (HF) range, at 13.56 MHz. The tag gets energized when placed in the reader's magnetic field, enabling its built-in circuitry to demodulate the data transmitted from the reader. The RFID reader keeps the magnetic field at the end of requests without any modulation to power the tag, enabling it to send back its reply to the reader. The tag chip sends back its response by internally modulating its input impedance (load modulation).

In order to design the circuit of a passive tag, its resonance frequency must match the reader operating frequency (13.56 MHz) since the tag obtains its power from the generated field by the reader. The power transfer from the reader to the tag and the communication range maximizes as the resonant frequency tunes to the carrier frequency. Hence, the tag antenna must be designed such that its equivalent inductance matches the internal tuning capacitance value to build a circuit resonating at the resonance frequency. The tuning capacitance is typically specified in the user manual by the manufacturer. The tuning capacitance for the ST25DV04K chip is 28.5 pF. We designed an air-cored, three-layer copper coil with a 5 cm radius and 34 American wire gauge (AWG) wire diameter as an antenna to be attached to the tag IC. The wires are taped into a plastic ring to fix the antenna inductance and unchanged over time. We also used a variable capacitor in parallel to the tag IC to adjust the equivalent capacitance to the inductance of the antenna. Supplementary Fig. 1 shows the RFID tag with its customized antenna and variable capacitor designed to operate at 13.56 MHz.

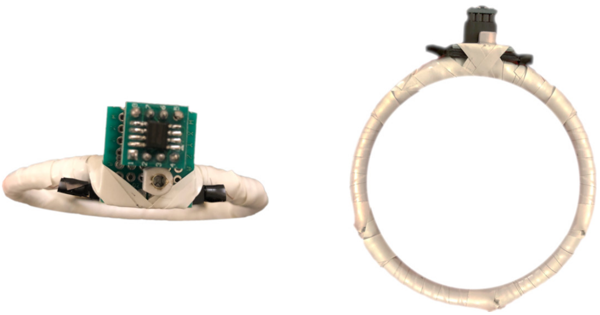

Supplementary Fig. 1. RFID transponder. HF tag with a customized antenna and variable capacitor is designed to operate at 13.56 MHz for experimental measurements.

Results

We measured received signal strength indicator (RSSI) data reported from the reader using FEIG's ISOStart software with an average sampling interval of 75 ms. The Kinect camera is also utilized to capture the motion of the reader and transponders while the reader records their RSSI values. The experiments are performed for 112 different movements with a duration of about 30 seconds for each sample. Both motion data recorded by Kinect and RSSI data are resampled with the time interval of 0.1 s. Supplementary Fig. 2 displays a sample including its measured RSSI and motions data. Using statistical measures, including correlation and R-squared (R^2), we studied the relationship between the RSSI data and the 3D distance of its corresponding tag antenna from the reader. The calculated results are then compared with those of magnetic induction (MI) experiments. Supplementary Fig. 3 presents the average R^2 and the correlation in all directions for both experiments. As the results indicate, the MI signal has a stronger relationship with its motion compared to a passive tag, and displacement in Z-direction (alignment of the reader antenna) has more effect on the MI signal than other directions. The outcomes are consistent with the results presented in this work, showing the accuracy of motion reconstruction by MI data is higher in the direction of reader surface norm.

In order to assess motion tracking using a passive tag's RSSI data recorded at the reader, we applied several machine learning regression models. Models are trained only on the measured data rather than synthetic signals since the MI model is not able to estimate power reflection from the tag to the reader using their motion data. The reason is that the load at the tag side, which is an essential input parameter of the MI model, varies over time. The amount of data available for model training and evaluation are restricted by the number of performed experiments, and therefore complex machine learning models are not considered. The limited number of training samples compared to that of the synthetic MI data can negatively impact the performance of regression models. The regressors are implemented and compared using the PyCaret library, and the

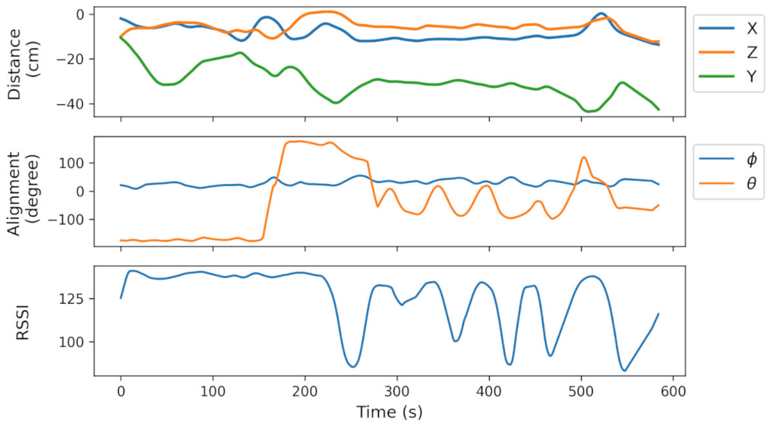

Supplementary Fig. 2. RFID measurement sample. An experimental RFID measurement including RSSI and motion data.

performance results are presented in Supplementary Table 1. Outcomes show that the signal strength delivered to the reader is not a useful feature in motion tracking.

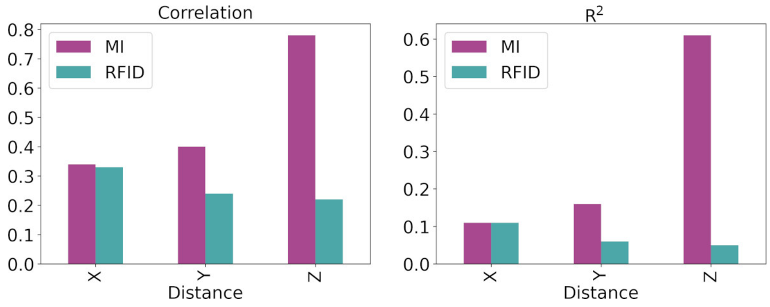

Supplementary Fig. 3. MI vs RSSI. Comparison of MI signal and RSSI of passive RFID in 3D motion tracking using statistical measures.

Supplementary Table 1. Performance of regression models in motion tracking using RSSI data measured by the RFID reader.

Model	RMSE	MAPE	R^2
LightGBM	**0.062**	0.137	**0.284**
RF	**0.062**	**0.134**	0.273
ET	0.063	**0.134**	0.26
KNN	0.063	0.135	0.249
MLP	0.064	0.145	0.225
LR	0.067	0.153	0.151
DT	0.085	0.169	0.102
