# Supplementary figures and images for: Wearable magnetic induction-based approach toward 3D motion tracking
Source: Sci Rep. 2021 Sep 23;11:18905. doi: 10.1038/s41598-021-98346-5 (PMC8460632; doi:10.1038/s41598-021-98346-5)

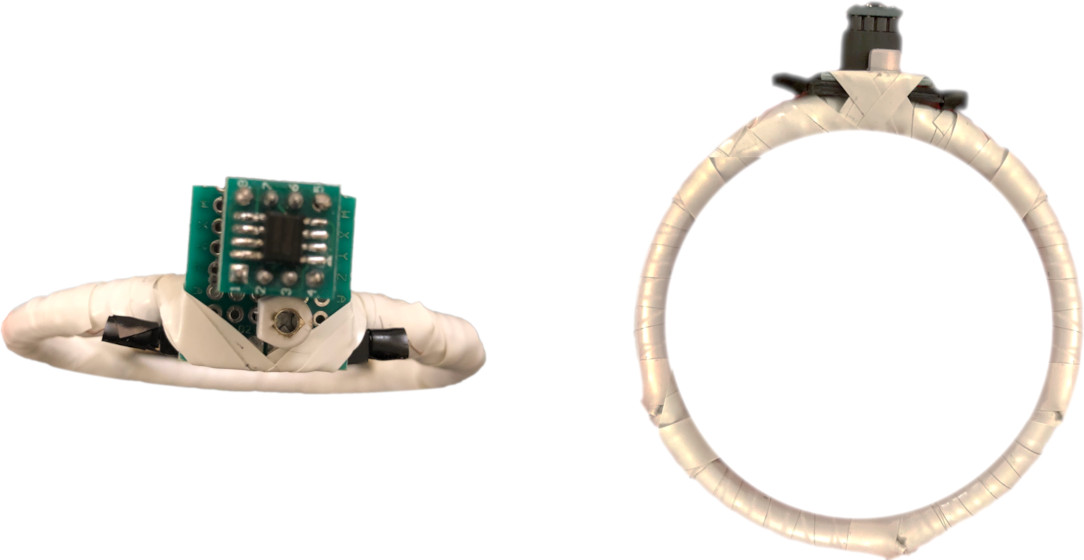

Supplement: Supplementary file 2 — Supplementary Information 2. [file 41598_2021_98346_MOESM2_ESM.jpg]

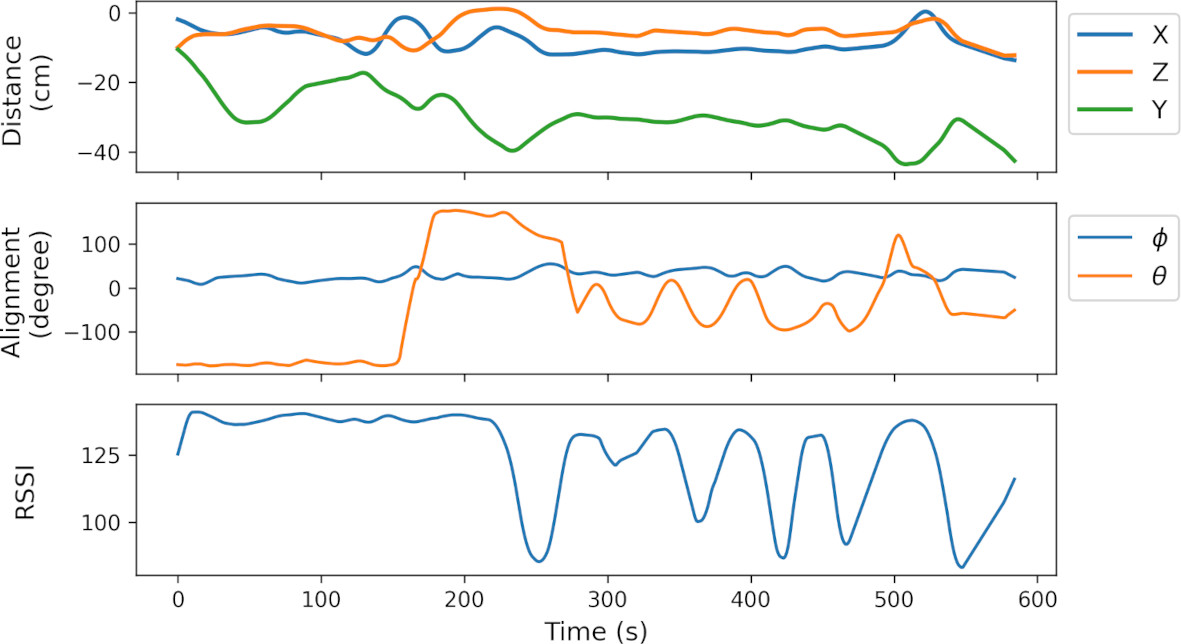

Supplement: Supplementary file 3 — Supplementary Information 3. [file 41598_2021_98346_MOESM3_ESM.jpg]

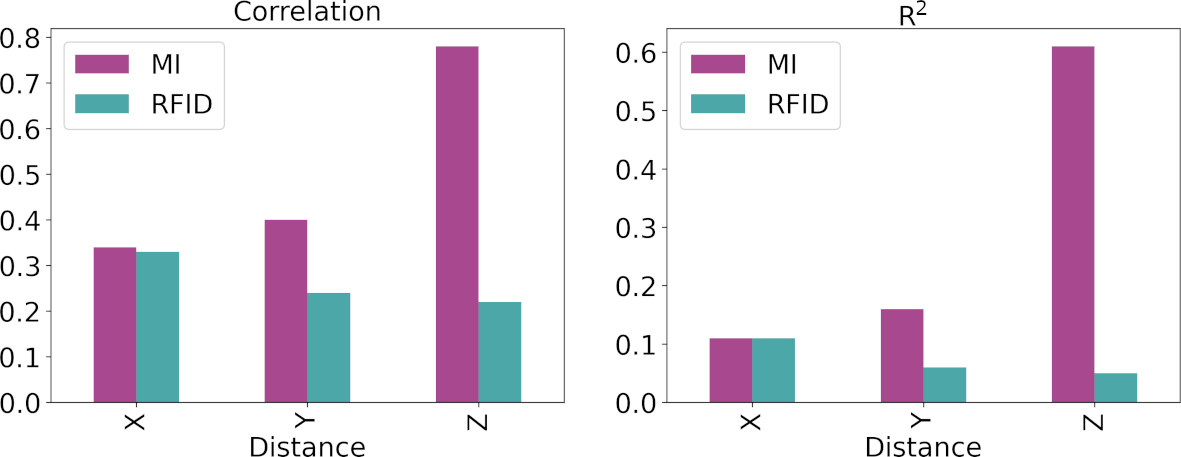

Supplement: Supplementary file 4 — Supplementary Information 4. [file 41598_2021_98346_MOESM4_ESM.jpg]
